# Engineered Self-Regulating Macrophages for Targeted Anti-inflammatory Drug Delivery

**DOI:** 10.21203/rs.3.rs-4385938/v1

**Published:** 2024-05-31

**Authors:** Molly Klimak, Amanda Cimino, Kristin Lenz, Luke Springer, Kelsey Collins, Natalia Harasymowicz, Nathan Xu, Christine Pham, Farshid Guilak

**Affiliations:** Washington University in St. Louis; Washington University in St. Louis; Washington University in St. Louis; Washington University in St. Louis; Washington University in St. Louis; Washington University in St. Louis; Washington University in St. Louis; Washington University in St. Louis; Washington University in St. Louis

**Keywords:** Cell therapy, rheumatoid arthritis, designer cell, synthetic biology

## Abstract

**Background:**

Rheumatoid arthritis (RA) is a systemic autoimmune disease characterized by increased levels of inflammation that primarily manifests in the joints. Macrophages act as key drivers for the progression of RA, contributing to the perpetuation of chronic inflammation and dysregulation of pro-inflammatory cytokines such as interleukin 1 (IL-1). The goal of this study was to develop a macrophage-based cell therapy for biologic drug delivery in an autoregulated manner.

**Methods:**

For proof-of-concept, we developed “smart” macrophages to mitigate the effects of IL-1 by delivering its inhibitor, IL-1 receptor antagonist (IL-1Ra). Bone marrow-derived macrophages were lentivirally transduced with a synthetic gene circuit that uses an NF-κB inducible promoter upstream of either the *Il1rn* or firefly luciferase transgenes. Two types of joint like cells were utilized to examine therapeutic protection *in vitro*, miPSCs derived cartilage and isolated primary mouse synovial fibroblasts while the K/BxN mouse model of RA was utilized to examine *in vivo* therapeutic protection.

**Results:**

These engineered macrophages were able to repeatably produce therapeutic levels of IL-1Ra that could successfully mitigate inflammatory activation in co-culture with both tissue engineered cartilage constructs and synovial fibroblasts. Following injection *in vivo*, macrophages homed to sites of inflammation and mitigated disease severity in the K/BxN mouse model of RA.

**Conclusion:**

These findings demonstrate the successful development of engineered macrophages that possess the ability for controlled, autoregulated production of IL-1 based on inflammatory signaling such as the NF-κB pathway to mitigate the effects of this cytokine for applications in RA or other inflammatory diseases. This system provides proof of concept for applications in other immune cell types as self-regulating delivery systems for therapeutic applications in a range of diseases.

## Background

Rheumatoid arthritis (RA) is a form of inflammatory arthritis that affects about 1% of the population, making it one of the most common autoimmune diseases [1, 2]. It is characterized by dynamic, episodic flares of systemic inflammation, resulting in morphological changes in the synovial membrane, bone destruction, and cartilage erosion that can lead to loss of joint function, pain and physical disability, and overall reduced patient quality of life [3–7]. Pro-inflammatory cytokines tumor necrosis factor-α (TNF-α), interferon-γ (IFN-γ), interleukin-1 (IL-1α, IL-1β), IL-6, and IL-17 have all been suggested to play critical roles in the pathogenesis of RA [8–12]. Secretion of these factors is initiated by resident cells such as chondrocytes, synoviocytes, and immune cells such as macrophages, which can make up 30–40% of the inflamed synovium and have been shown to play a pivotal role in RA. Macrophages can amplify underlying inflammation both locally and systemically through the overexpression of proinflammatory cytokines, growth factors, matrix metalloproteinases and chemoattractants in the synovial membrane and at the cartilage-pannus junction [6, 13–20]. In particular, TNF-α and IL-1 signaling from macrophages triggers the nuclear factor kappa light chain enhancer of activated B cells (NF-κB) pathway, which perpetuates the inflammatory environment of RA through a host of downstream cascades including the recruitment of additional immune cells, induction of T-cell differentiation, and increased bone resorption [21–23].

Biologics that antagonize inflammatory cytokines are the cornerstones of current RA treatment [4, 24–31]. While many of these therapies are effective in treating a subset of people with RA, they are often delivered continuously at high doses, which can predispose patients to significant off-target effects, including increased susceptibility to infection and limited tissue regeneration and repair [7, 32, 33]. Additionally, as cytokine production in RA patients occurs in flares both on daily scales, with production peaking in the early hours of the morning, and on more long-term scales, with flares that can vary in duration from weeks to months, most patients do not require a traditional, consistent high dose treatment [34]. Therefore, developing advanced biological delivery strategies with the ability to deliver drugs in a controlled, localized, and autoregulated manner will lead to a more targeted and effective treatment of inflammation in RA.

Cell-based therapies have broad potential for use in autoimmune and inflammatory diseases. Recent work in immunoengineering offers a new perspective utilizing cell therapies and genetic engineering approaches where cells can be engineered to express inducible transgenes for dynamic, microenvironment-level control of cell response, eliminating the need for systemic and repeated, high-dose therapies [35–39]. Due to their prominent role in the inflammatory cascade and pathology of RA, macrophages represent a potential target for interrupting the perpetuating production of proinflammatory factors. Furthermore, as macrophages innately home to microenvironments that generate inflammatory chemokines, they provide a unique opportunity as a cell-based drug-delivery systems that can target delivery to affected areas, a major limitation in current therapies [4, 15, 40, 41].

**Figure 1:**
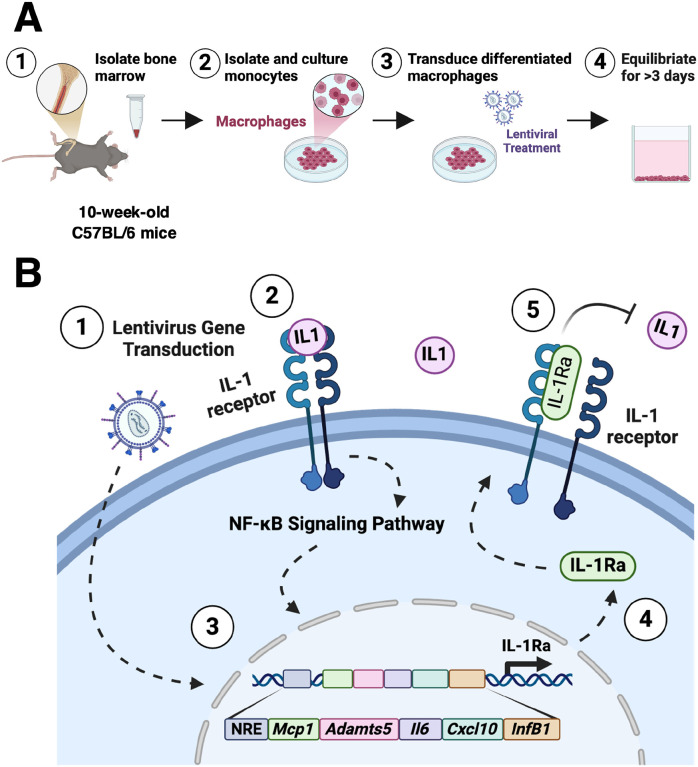
Overview of the therapeutic macrophages and inducible NF-κB lentiviral synthetic promoter. **(A)** Overview of the generation of engineered macrophages from the bone marrow of C57BL/6 mice following lentiviral transduction of the inducible NF-κB lentiviral circuit. **(B)** Response of IL-1 signaling in the therapeutic NF-κB-IL-1Ra circuit. (1) Following lentiviral transduction, (2) IL-1 signaling through the IL-1 receptor initiates a cascade leading to (3) nuclear translocation and increased transcriptional activity of NF-κB, activating an inflammatory transcriptional program and induces *Il1rn* expression, (4) driving production of IL-1Ra that acts as an IL-1 receptor antagonist (5) inhibiting activation of the NF-κB inflammatory cascade. An NRE was inserted upstream of the promoter to reduce background signal. To characterize activation dynamics, a separate inducible NF-κB circuit, denoted as ‘LUC’, was designed to drive the firefly luciferase gene downstream of the same promoter; IL-1Ra: interleukin-1 receptor antagonist; NF-κB: nuclear factor kappa-light-chain-enhancer of activated B cells; NRE: negative regulatory element.

The goal of this work was to develop a genetically-engineered macrophage capable of delivering an anti-inflammatory biologic drug in response to inflammatory signaling. To accomplish this goal, we engineered macrophages (Fig. 1A) to contain a synthetic gene circuit that drives the production of anti-inflammatory factor IL-1Ra in response to the activation of canonical NF-κB recognition motifs (Fig. 1B) [42]. To fully characterize the ability of an engineered macrophage as a viable cell therapy, we examined their potential to 1) respond to inflammatory stimuli in dose dependent manner; 2) autoregulate in response to ‘flares’; 3) modulate their local environment to reduce downstream effects of inflammation; and 4) home to sites of inflammation to decrease RA severity. We initially tested these responses *in vitro* using a co-culture system with either murine induced pluripotent stem cell (miPSC)-derived cartilage pellets or primary synovial fibroblasts, as these are two key cell populations that are dysregulated in the presence of arthritis [43–45]. The therapeutic NF-κB-IL-1Ra macrophages were able to attenuate inflammatory propagation and matrix degradation in both cell types. To test the ability of our therapeutic system to home and reduce inflammation as well as RA severity, we utilized both an acute joint injury model and a short-term, flare-like model of RA [46–49]. Engineered macrophages were able to home to and persist at the site of injury while mitigating inflammation and disease severity. Here, we established an engineered macrophage NF-κB-inducible biologic delivery system that allowed for controlled, self-regulated production of IL-1Ra in response to NF-κB activation that effectively mitigated inflammation and hallmarks of RA.

## Methods

### Overall strategy.

The overall goal of this work was to create a macrophage-based system that is activated by inflammatory cytokines and engineered to express anti-inflammatory biologic drugs downstream of a synthetic NF-κB-responsive promoter, providing a negative feedback system that blocks the perpetuation of macrophage inflammatory signaling (Fig. 1A). Here, we specifically tested the ability of these engineered macrophages to modulate their production of inflammatory mediators, protect miPSC derived cartilage from inflammatory degradation, and remain viable in a mouse model for up to 7 days.

### Macrophage differentiation.

L929-conditioned media was generated by culturing L929 cells, a normal fibroblast cell line that secretes macrophage colony-stimulating factor-1 (M-CSF) from subcutaneous connective tissue of mouse (< passage 3) for 7 days in Dulbecco’s modified Eagle’s medium/Nutrient Mixture F-12 (DMEM/F12), 10% fetal bovine serum (FBS), 1% penicillin/streptomycin (P/S). Media was collected, filtered, and stored at −20°C until use. Bone marrow derived macrophages (BMDM) were generated from the bone marrow isolated from long bones in C57BL/6 mice. Bone marrow was incubated with red cell lysis buffer and strained with a 40 μm strainer prior to collection of isolated cells. Cells were then cultured for 10 days in Dulbecco’s modified Eagle’s medium-high glucose (DMEM-HG) with 10% FBS, 1% P/S and 30% L929-conditioned media prior to testing.

### Generation of iPSC derived cartilage.

Murine iPSCs (miPSCs), generated from tail fibroblasts from adult C57BL/6 mice and validated for pluripotency as described by Diekman et al., were maintained on mitomycin C-treated mouse embryonic fibroblasts (MEFs) (Millipore). miPSCs were differentiated toward a mesenchymal state using a high-density micromass culture in DMEM-HG with 1% recombinant human insulin, human transferrin, and sodium selenite (ITS+, Corning), 1% minimum essential medium (MEM) non-essential amino acids, 1% P/S, 55 μM β-mercaptoethanol, 50 μg/mL L-ascorbic acid, and 40 μg/mL L-proline. Medium was additionally supplemented with 50 ng/mL bone morphogenic protein-4 (BMP-4, R&D Systems) and 100 nM dexamethasone on the third and fifth day of micromass culture. After 15 days of culture, micromasses were dissociated with pronase and collagenase type II and plated on gelatin-coated dishes in expansion medium as MSC-like pre-differentiated iPSCs. Expansion medium consisted of DMEM-HG with 10% FBS, 1% ITS+, 1% MEM non-essential amino acids, 1% P/S, 55 μM β-mercaptoethanol, 50 μg/mL L-ascorbic acid, 40 μg/mL L-proline, and 4 ng/mL basic fibroblast growth factor (bFGF, R&D Systems). After expansion, these cells were pelleted at 250,000 cells per pellet and differentiated into 3D cartilage pellets over 21 days in DMEM-HG with 1% ITS+, 1% MEM non-essential amino acids, 1% P/S, 55 μM β-mercaptoethanol, 50 μg/mL L-ascorbic acid, 40 μg/mL L-proline, and 10 ng/mL transforming growth factor-β3 (TGF-β3, R&D Systems).

### Lentiviral production and cell transduction.

A standard protocol was followed to produce second-generation packaged vesicular stomatitis virus glycoprotein pseudotyped lentivirus. HEK293T cells were co-transfected with an expression transfer vector, second-generation packaging plasmid psPAX2 (No. 12260; Addgene), and an envelope plasmid pMD2.G (No. 12259; Addgene) by calcium phosphate precipitation. Three separate expression transfer vectors were used to generate IL-1Ra-, Luciferase-, and GFP-producing lentiviruses. Lentiviruses were stored at − 80°C until use. During transduction, media was supplemented with polybrene and the desired number of viral particles to achieve an average multiplicity of infection (MOI) 40–50 for engineered macrophages. Viral titers were conducted via quantitative real-time polymerase chain reaction (qRT-PCR) to determine the number of lentiviral DNA copies integrated into the genome of transduced BMDMs and MSC-like cells at their respective MOI.

### Culture methods for in vitro inflammatory challenges.

Following viral transduction of BMDM and differentiation of chondrocyte pellets, cells were exposed to inflammatory cytokines *in vitro* and evaluated in culture medium without TGF-β or dexamethasone. Engineered macrophages were subject to varying concentrations of IL-1α or IL-1β (R&D Systems) to assess cellular responses in monoculture and compared to non-transduced BMDM in the same conditions. Macrophages were seeded into experimental wells at concentrations of 370,000 cells per well for 6-well co-cultures and at 185,000 cells per well for 24-well co-cultures at least 3 days prior to experiments to reduce inflammatory activation that may be caused by seeding cells. Direct and transwell co-cultures of chondrocyte pellets with engineered macrophages were maintained in chondrocyte pellet medium. Macrophage culture medium was replaced with chondrocyte pellet medium (excluding TGF-β and dexamethasone) 24 hours before beginning co-cultures to reduce potential inflammatory activation due to the differences in media components.

Prior to co-culture, chondrocyte pellets were pre-treated with IL-1β for 24 hours. At the start of co-culture, cells were introduced into non-treated, basal medium or IL-1β-treated medium for 48 hours (Fig. 3). For direct co-cultures, chondrocyte pellets were added directly into the media of wells with engineered macrophages. For transwell co-cultures, engineered macrophages were seeded into wells, and chondrocyte pellets were added to 4 μm-pore transwell inserts (Corning) so that culture medium was shared but macrophages and pellets were not in direct contact. Two pellets were introduced per co-culture to match experimental conditions across analyses approximating a 1:1 ratio of chondrocytes to macrophages.

### Boyden cell migration assay.

Monoculture BMDM were collected by trypsinization and incubated in serum starvation medium (DMEM-HG with 1% P/S) for 1 hour. Then, BMDM were resuspended in DMEM-HG at a concentration of 500,000 cells/mL. In the Boyden chamber, chemoattractants were added to the bottom chamber of the Neuro Probe 48-well chemotaxis chambers, and 50,000 BMDM were introduced into the top chamber. Polycarbonate PFB filters (Neuro Probe) with 8-μm pores were used to separate top and bottom chambers. Chambers were incubated at 37°C for 16 hours. Migrated cells on the filter were fixed in methanol, stained with VECTASHIELD mounting media with DAPI (Vector Laboratories), and imaged by fluorescence microscopy (VS120, Olympus).

### Phagocytosis assay.

The Phagocytosis Assay Kit (Cayman Chemical Company) was performed according to the manufacturer’s instructions. Engineered or non-transduced macrophages in monoculture were cultured for 24 hours in basal macrophage medium, medium treated with 100 ng/mL interferon-γ (IFN-γ) and 1 ng/mL lipopolysaccharide (LPS), medium treated with 10 ng/mL IL-4 and 10 ng/mL IL-13, or medium treated with 1 or 10 ng/ml of IL-1β. After 24 hours, macrophages were incubated in medium containing latex beads coated with FITC-labeled rabbit IgG (1:400) or in control medium for 4 hours. Excess beads were removed by washing with PBS, and cells were scraped in 1 mL of assay buffer into a polypropylene tube. Cells were fixed in 4% paraformaldehyde (PFA), stained with CD14-PE-Cy7 as a macrophage marker and SytoxBlue to identify dead cells, and analyzed by flow cytometry.

### Western blot.

Engineering macrophages in monoculture were rinsed with PBS, incubated on ice in RIPA buffer for 5 minutes, collected, and centrifuged at 14,000 × g for 10 minutes to pellet the cellular components. Proteins were denatured in a Laemmli sample buffer (Biorad) at 95°C for 5 minutes, separated by standard SDS/PAGE using 7.5% polyacrylamide gels, and transferred to a PVDF membrane. Membranes were rinsed with PBS/Tween20 buffer and blocked at room temperature for 1 hour with 5% milk in PBS/Tween20. After rinsing, membranes were probed with primary antibodies (sheep anti-CD206; rabbit anti-iNOS; 1:1000 rabbit anti-GAPDH) at 4°C overnight, rinsed, and then incubated with secondary antibodies at 1:1000 (goat pAb to sheep IgG for CD206; mouse pAb to rabbit IgG for iNOS and GAPDH) at room temperature for 1 hour. Blots were developed with Pierce ECL Western Blotting Substrate and imaged with a ChemiDoc XRS.

### Circuit Activity Assays.

Circuit activity with or without inflammatory challenge (IL-1α or IL-1β) at designated timepoints was assessed using the Bright-Glo Luciferase Assay System (Promega) according to the manufacture’s recommendations, and luminescence readings were recorded (BioTek Cytation 5). To monitor circuit activity semi-continuously, engineered macrophages were cultured in phenol-free medium with 125 μM D-luciferin (Promega) in a temperature and CO_2_-controlled system, and relative luciferase activity was quantified based on luminescence readings every 15 minutes (BioTek Cytation 5). Following 24-hour baseline measurements (n = 6–8) in non-treated medium, culture medium was treated with IL-1β to induce inflammatory activity. Luciferase activity is reported as relative luminescence with respect to non-IL-1β treated control activity normalized to average 24-hour baseline measurements.

### Histology and immunohistochemistry.

After fixation in 10% neutral buffered formalin for 24 hours, pellets were paraffin-embedded, sectioned at 8 μm thickness, and stained using a standard protocol for sulfated glycosaminoglycans (sGAG) and collagenous matrix with Safranin-O and fast green, respectively, while hematoxylin was used as a nuclear counterstain. Immunohistochemistry (IHC) was used to detect type II collagen (COL2A1). After sectioning, samples were incubated in proteinase K to retrieve antigens followed by 3% H_2_O_2_ in methanol to quench peroxidase. Samples were blocked using 10% normal goat serum and then incubated with a mouse-generated anti-COL2A1 primary antibody (Iowa II-II6B3-s) followed by a biotinylated goat anti-mouse IgG secondary antibody (Abcam #150771). Secondary antibodies were enzyme conjugated with HRP and incubated with AEC substrate (Invitrogen). Samples were counterstained with Vector Hematoxylin QS (Vector Laboratories). Histological and IHC samples were imaged by brightfield microscopy (VS120, Olympus).

### Biochemical analysis.

Pellets were washed with PBS and stored at −20°C until processing. Pellets were digested in 125 μg/mL of papain overnight at 65°C. DNA content was measured with PicoGreen assay (Invitrogen), and total sGAG content measured using a 1,9-dimethylmethylene blue (DMMB) assay at 525 nm wavelength.

### Gene expression.

RNA from macrophages and pellets was isolated according to the manufacturer’s recommendations (Norgen Biotek). Prior to RNA isolation, chondrocyte pellets were homogenized using a miniature bead beater. 100–150 ng of RNA was reverse transcribed using SuperScript VILO complementary DNA (cDNA) master mix (Invitrogen). qRT-PCR was performed using Fast SyBR Green master mix (Applied Biosystems) on a QuantStudio (ThermoFisher). For qRT-PCR, 10 ng of cDNA per reaction with primer concentrations at 10 μM (Integrated DNA Technologies, Supplementary Table 1) were analyzed. Reactions were performed in technical duplicates for each analyzed gene. Fold changes were calculated using the ΔΔCT method relative to no treatment control samples.

### Enzyme-linked immunosorbent assay.

Culture media was collected and stored at −20°C until analysis. IL-1Ra concentration was measured with DuoSet enzyme-linked immunosorbent assay (ELISA) specific to mouse IL-1Ra (R&D Systems). Each sample was assessed in technical duplicates, and absorbance was measured at 450 and 540 nm.

### Griess assay.

Culture media was collected and stored at −20°C until analysis. The concentration of nitrite (NO_2_) in culture media was quantified in technical duplicates by the Griess reagent (Promega) according to the manufacturer’s instructions.

## Mouse experiments

### Joint acute injury model and cell delivery.

16-week C57BL/6 mice underwent no surgery (n = 3) as a control or destabilization of the medial meniscus surgery (n = 3). Mice were allowed four days for recovery prior to administration of cells. Mice received macrophages lentivirally transduced with constitutive GFP and the NF-kB circuit at a dose of 0.5 million cells in 50 mL of PBS via intraarticular injection.

### K/BxN model of inflammatory arthritis and cell delivery.

The model and methods were adapted from previous work [49]. Prior to delivery, engineered macrophages were additionally transduced to constitutively express luciferase for all animal studies. C57BL/6 (7-week-old) mice were challenged with 180 μL of K/BxN serum delivered by retro-orbital injection to induce spontaneous arthritis. 24 hours following disease initiation, mice were intraperitoneally (i.p.) injected with either PBS or 5 million LUC or IL-1Ra macrophages in 200 μL of PBS (n = 9–15/group). Disease activity (clinical score and ankle thickness) and macrophage presence (IVIS) were assessed daily for one week. Mice were then sacrificed, and further IVIS imaging of paws, kidney, lungs, liver, and spleen was conducted. Hind paws were evaluated for bone erosions using microCT (Bruker SkyScan 1176). Levels of IL-1Ra in serum were assessed by a Quantikine ELISA kit (R&D Systems). All procedures were approved by the Institutional Animal Care and Use Committee at Washington University in St. Louis. The clinical score was assessed on a scale of 0 to 3 (0, no swelling or erythema; 1, slight swelling or erythema; 2, moderate erythema and swelling in multiple digits or entire paw; and 3, pronounced erythema and swelling of entire paw; maximum total score of 12). The change from baseline in ankle thickness was determined daily by dial calipers, and an average change in the ankle thickness was determined for each mouse from the two hind paw measurements.

### Pain and behavioral testing.

Algometry was performed using a pressure-based analgesiometer (SMALGO, Bioseb, Vitrolles, France) by applying a progressive force over the ankle joint, and the stimulation was increased until a reaction (shudder or vocalization) from the animal was obtained. The maximum force was then automatically recorded, analyzed, and reported as pressure-pain hyperalgesia. To determine tactile allodynia, an electronic Von Frey paw assay was used (electronic von Frey anesthesiometer, IITC Inc., Life Science Instruments, Woodland Hills, CA, USA). Mice were placed in acrylic cages (12 cm by 10 cm by 17 cm high) with a wire grid floor and a tip was applied against the central edge of the animal hind paw where the intensity of the stimulus was automatically recorded when the paw was withdrawn. The stimulation of the paw was repeated for three measurements.

### IVIS Luminescence.

In vivo bioluminescence imaging was conducted using an IVIS 50 (PerkinElmer, Waltham, MA; Living Image 4.3.1, 5 min exposure, 2 bin, FOV12cm, f/stop1, open filter). Hair was removed from the area before imaging. Mice were weighed each day, injected intraperitoneally with D-luciferin (150mg/kg in PBS; Gold Biotechnology) and imaged 10 minutes later using isoflurane anesthesia (2% vaporized in O_2_). Total photon flux (photons/sec) was measured from two fixed regions of interest (ROIs) - the abdomen and joint - using Living Image 2.6.

### Tissue sectioning and immunofluorescence.

Following dissection, all tissues were incubated at room temperature in a 15% sucrose in PBS solution for 1 hour followed by a 30% sucrose in PBS solution for 1 hour. Joint Injury Model: Tissues were embedded in Tissue-Tek OCT compound, snap frozen on dry ice, and stored at −80°C until sectioning. Frozen tissues were cryosectioned at 8 μm thickness. Tissue was blocked at room temperature in 2.5% normal goat serum for 1 hour. Cells were incubated with CD14-PE or CD11b-PE (1:100) for 1 hour at room temperature, washed with PBS/Tween, and mounted with VECTASHIELD mounting media with DAPI (Vector Laboratories). Images were captured using a Zeiss confocal laser scanning microscope.

### K/BxN Model

Paws were harvested on day 7 after serum transfer, fixed in 4% paraformaldehyde, decalcified in EDTA solution, embedded in paraffin, and sectioned at thickness 5 μm. Sections were stained with hematoxylin and eosin (H&E) or toluidine blue. Inflammatory cells infiltrating the synovial lining and the joint cavity were enumerated in 8 to 10 random fields per section using H&E images acquired at ×400 magnification. Proteoglycan loss in the cartilage was scored on toluidine blue–stained sections on a scale from 0 to 3, ranging from fully stained cartilage (score, 0) to fully unstained cartilage (score, 3), as previously described [49]. Scoring was performed by an observer blinded to the treatment.

GFP/engineered macrophage presence in the mouse paw was detected using immunohistochemistry. Paraffin sections of scaffold were rehydrated, washed, and incubated for 20 min at 37°C with proteinase K diluted in 10 mM tris-HCl (pH 8.0) for antigen retrieval. The slides were then treated with 3% peroxidase H_2_O_2_ in methanol and blocked with 5% goat serum for 1 hour at room temperature. Sections were then stained with a primary GFP polyclonal antibody (A-11122, Life Technologies) overnight at 4°C before staining with an anti-rabbit secondary antibody at room temperature for 75 minutes. Sections were then labeled with DAB/hematoxylin and imaged.

### Flow cytometry.

Flow cytometry was performed in vitro on cultured macrophages and *in* vivo on cells dissociated from isolated tissues and fluids to examine the expression of typical macrophage and MSC cell markers to confirm presence as well as activation. Following dissection, cells from the spleen and liver were prepared by mincing the tissues with a syringe plunger through a 70 μm cell strainer, washing isolated cells with excess PBS, incubating pelleted cells in cold RBC lysis buffer, and then resuspending the cell pellet in PBS. Peritoneal cells were collected by administration and collection of 10 mL of PBS from the peritoneal space. All samples prior to staining were passed through a 40 μm strainer to remove debris and clumps and blocked with Fc receptor antibody CD16/32 to prevent non-specific Fc receptor-mediated antibody binding before staining. Dead cells were stained with SytoxBlue, and doublets and cellular debris were excluded through Flow Jo analysis.

### MicroCT analysis of bone erosion.

To measure bone morphological changes, hind paws were scanned by microCT (SkyScan 1176, Bruker) with a 9-μm isotropic voxel resolution at 50 kV, 500 μA, 980-ms integration time, 3 frame averaging, and 0.5-mm aluminum filter to reduce the effects of beam hardening. Images were reconstructed using NRecon software (with 20% beam hardening correction and 15-ring artifact correction). The parameters reported are as follows: BS/BV, a bone fraction (BV/TV), and BMD (in g/cm3).

## Statistical analysis.

### In vitro assays

Statistical analysis was performed with Graphpad Prism using analysis of variance (ANOVA) with no treatment as the control (α = 0.05). A one-way ANOVA (monoculture data) or two-way ANOVA (all co-culture data) with Tukey’s HSD post hoc test was used.

### Mouse studies

Sample size was determined on the basis of a mean clinical score of 10 ± 2 for K/BxN mice. Normality was assessed by group using a Shapiro-Wilk test. Outcomes were evaluated by two-way Student’s t test or one-, two-, and three-way repeated measures analysis of variance (ANOVA) with a Geisser-Greenhouse correction to account for sphericity. Tukey’s post hoc test was used to assess differences between groups, treatments, time, or a combination of those factors. All disease activity, pain testing, and in vivo imaging assessments were performed blinded.

## Results

### Engineered macrophages with the NF-κB synthetic promoter dynamically autoregulate in response to inflammation

**Figure 2:**
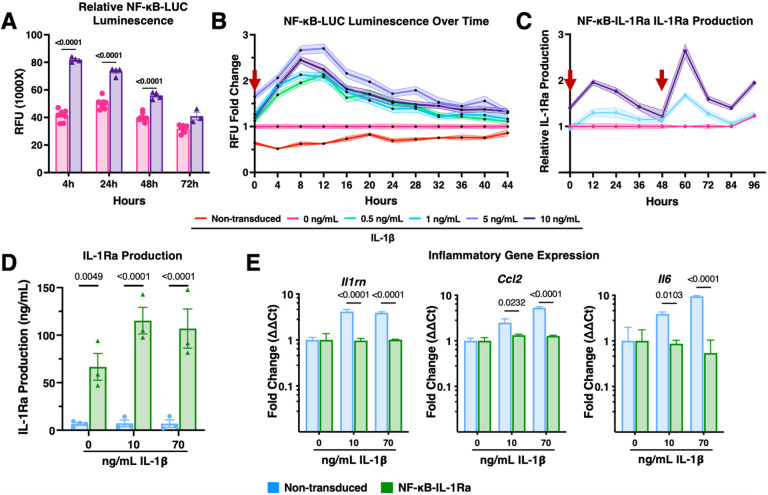
Engineered macrophages with the NF-κB synthetic promoter dynamically autoregulate in response to inflammation in monolayer. **(A)** NF-κB-LUC macrophages demonstrate circuit activation by 4 hours which is resolved by 72 hours following treatment with IL-1β (n=5). **(B)** Relative luminescence normalized to a 24-hour baseline by NF-κB-LUC macrophages increases in response to higher concentrations of IL-1β over time (n=12); red arrow represents addition of stimulus. **(C)** NF-κB-IL-1Ra macrophage production of IL-1Ra, as measured by ELISA, following stimulation with 1 or 10 ng/ml of IL-1β at 0 and 48 hours normalized to a non-treated control (n=5); red arrow represents addition of stimulus. **(D)** ELISA for IL-1Ra production from non-transduced and NF-κB-IL-1Ra macrophages in response to no treatment, 10 or 70 ng/mL IL-1β demonstrates significant production of IL-1Ra in NF-κB-IL-1Ra macrophages (n=4). **(E)** Gene expression of NF-κB-IL-1Ra BMDMs in monoculture in reference to non-transduced, non-treated BMDMs when challenged with 10 or 70 ng/mL IL-1β for 48 hours normalized to *Gapdh* and *R18s* (n=4) demonstrated significant reduction in NF-κB responsive genes by NF-κB-IL-1Ra macrophages for both medium and high doses. Data was assessed using a one or two-way ANOVA with Tukey’s post hoc test; values represent mean with standard error of the mean (SEM) (n = 4–12); RFU: relative luminescence unit; IL-1β: Interleukin-1 beta.

To evaluate the activation of our inflammation-responsive circuits, murine bone marrow-derived macrophages (BMDM) were lentivirally transduced and assessed using the reporter NF-κB-LUC circuit, where a firefly luciferase transcriptional reporter was inserted in place of the *Il1rn* transgene, allowing visualization of circuit activity (Figure 2A). Luminescence was examined at 4-, 24-, 48- and 72-hours following treatment with either 0 or 10 ng/mL of IL-1a or IL-1b. NF-κB-LUC macrophages demonstrated a two-fold activation over untreated controls in response to both IL-1a or IL-1b treatment at 4 hours, which resolved by 72 hours (Figure 2A, Supplemental Figure 1A). To further characterize activation kinetics, NF-κB-LUC macrophages were cultured with luciferin in addition to IL-1b at a range of doses from physiologic (0.5 ng/mL) to supraphysiologic (10 ng/mL) and compared to a non-transduced control. Luminescence readings were taken every 15 minutes until the signal returned to baseline (Figure 2B). Circuit activation peaked around 12 hours for all IL-1b doses, with 0.5 ng/mL exhibiting the lowest and 5 ng/mL the highest luminescent signal.

To examine the ability of the NF-κB-IL-1Ra system in macrophages to reactivate in response to ‘flares’, we performed two iterative stimulations 48 hours apart with either 0, 1, or 10 ng/mL of IL-1b (Figure 2C). Macrophages responded to both stimulations in a dose-dependent manner, peaking once more around 12 hours after stimulation, and returning to baseline by 48 hours after stimulation with significantly higher amounts of IL-1Ra produced in response to 10 ng/mL of IL-1b than 1 ng/mL IL-1b at each timepoint. Overall, these results suggest that both the NF-κB-LUC and NF-κB-IL-1Ra circuits operate according to a similar kinetic profile.

The range of response of NF-κB-IL-Ra macrophages to inflammatory stimuli was then evaluated where macrophages were treated with a variety of inflammatory cytokines typically elevated in RA (IL-1b, IL-1a, and TNF-a) for 48 hours and compared to a non-transduced control. While non-transduced macrophages produced low levels of IL-1Ra independent of dose, NF-κB-IL-1Ra macrophages demonstrated significant production of IL-1Ra (up to 6 ng/mL) in response to IL-1b (Figure 2D). Gene expression analysis demonstrated significant downregulation in expression of inflammation-related genes, *Ccl2* and *Il6*, in NF-κB-IL-*1Ra* macrophages compared with a non-transduced control (Figure 2E and Supplemental Figure 1B). Furthermore, *Il1rn* gene expression was downregulated by 48 hours (Figure 2E). Notably, NF-κB-IL-1Ra macrophages were also able to mitigate inflammatory activation in response to treatment with IL-1a at all tested concentrations and TNF-a at 5 ng/mL, suggesting that this system could respond to multiple forms of NF-κB mediated inflammation (Supplemental Figure 1B).

### Engineered macrophages maintain their polarization and function when transduced with gene circuits

To determine if either circuit affected macrophage phenotype or function, NF-κB-LUC and NF-κB-IL-1Ra macrophages were stimulated then subsequently analyzed. Macrophages expressing either the NF-κB-LUC or NF-κB-IL-1Ra circuit were treated with 0, 1, or 10 ng/mL of IL-1β for 48 hours and compared to a non-transduced control. Western blot analysis displayed increased CD206 expression in NF-κB-IL-1Ra macrophages while both non-transduced and NF-κB-LUC macrophages displayed a small decrease in expression compared to non-treated controls (Supplemental Figure 1C). Likewise, the inflammatory marker nitric oxide synthase 2 (NOS2) decreased as the level of IL-1b treatment increased in NF-κB-IL-1Ra macrophages, while the reverse was observed for both non-transduced and NF-κB-LUC macrophages. To examine the effect on phagocytotic capacity, non-transduced, NF-κB-LUC, and NF-κB-IL-1Ra macrophages underwent cytokine stimulation for 24 hours prior to a short incubation with latex beads. Quantification of bead uptake by flow cytometry showed no differences between macrophage subtypes in response to stimulation (Supplemental Figure 1D). To further test macrophage function, migration in response to both cytokine and chemokine signals was examined for non-transduced, NF-κB-LUC, and NF-κB-IL-1Ra macrophages using a Boyden chamber migration assay. In the absence of a chemoattractant, there was little migration observed (Supplemental Figure 1E). All macrophages exhibited a dose-dependent migration in response to IL-1β as well as conditioned media from chondrocytes treated with IL-1β, which elicited the highest level of migration.

### NF-κB-IL-1Ra macrophages protect against cartilage matrix degradation in response to IL-1β

To determine the ability of engineered macrophages to mitigate inflammation in a joint-like environment, we utilized a direct and an indirect transwell co-culture model with tissue engineered 3D cartilage constructs as a representative model of articular cartilage that is degraded in the presence of inflammatory signals. Initially, murine induced pluripotent stem cells (miPSCs)-derived articular cartilage-like pellets were generated following a 48-day protocol for optimized chondrogenic differentiation [45, 50]. Cartilage pellets were pre-treated with 0 or 1 ng/mL of IL-1β for 24 hours to mimic the inflammatory environment of RA (Supplemental Figure 2A). Pellets were then placed in direct co-culture with either non-transduced or NF-κB-IL-1Ra macrophages and treated with 0 or 1 ng/mL IL-1β in the co-culture media. All macrophages produced significant amounts of IL-1Ra in response to IL-1β as compared to non-treated controls (Supplemental Figure 2B). Cultures with NF-κB-IL-1Ra macrophages produced significantly higher levels of IL-1Ra than non-transduced macrophage co-cultures, producing 100 pg of IL-1Ra per 10^3^ macrophages in co-culture by 48 hours in response to both 0 and 1 ng/mL of IL-1β. As a measure of overall inflammation, the level of nitric oxide (NO) in the culture media was assessed but exhibited no significant differences between groups (Supplemental Figure 2C). Histological analysis of Safranin-O staining for sulfated glycosaminoglycans (sGAGs), one of the primary components of cartilage ECM, showed rich staining in non-treated pellets that was reduced following treatment with 1 ng/mL IL-1β but was maintained in co-cultures with NF-κB-IL-1Ra macrophages (Supplemental Figure 2D). However, there was no significant difference in sGAG/DNA content between non-transduced and NF-κB-IL-1Ra macrophage co-cultures (Supplemental Figure 2E). As expected, Acan and *Col2a1* were reduced, and inflammatory genes *Ccl2*, *Pges2, Il6*, and *Mmp13* were increased following IL-1β treatment when co-cultured with non-transduced macrophages (Supplemental Figure 2F). Co-cultures with NF-κB-IL-1Ra macrophages, however, offered substantial protection against both loss of cartilage-associated gene expression and activation of inflammatory gene expression.

**Figure 3:**
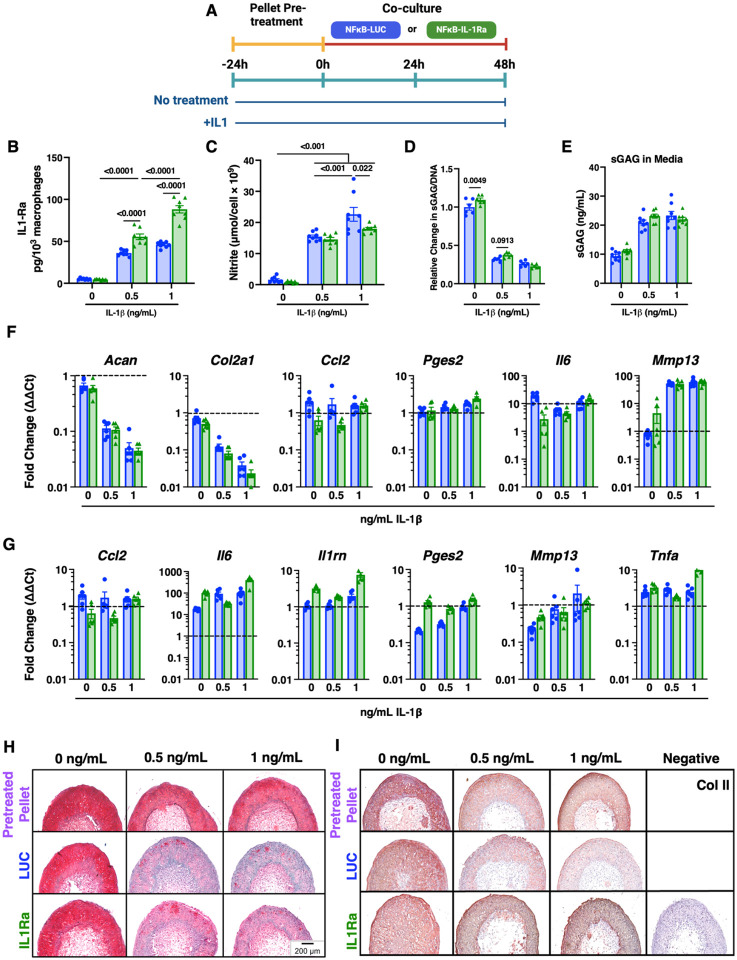
NF-κB-IL-1Ra macrophages offer moderate chondroprotection against inflammatory mediators and inflammatory driven matrix degradation in response to IL-1β. **(A)** Timeline of co-culture experimental conditions. miPSC derived cartilage pellets were initially pre-treated with 0, 0.5 or 10 ng/mL of IL-1β prior to co-culture for 24 hours. Pellets were placed into co-culture with transduced macrophages and re-treated with 0, 0.5, or 1 ng/mL of IL-1β for 48 hours in a 24 well Transwell plate (n=3–6). **(B)** Total IL-1Ra in co-culture over 48 hours, as measured by ELISA normalized to cell number, following treatment with IL-1β. **(C)** Concentration of nitric oxide secreted into the media normalized to cell number following treatment with IL-1β. **(D)** Amount of sGAG per pellet normalized to DNA content. **(E)** Total amount of sGAG secreted into the media. **(F)** Gene expression of miPSC derived cartilage pellets in co-culture in reference to non-treated control when challenged with IL-1βfor 48h normalized to *Gapdh* and *R18s* (n=4–6). **(G)** Gene expression of engineered macrophages in co-culture in reference to non-treated control when challenged with IL-1βfor 48h normalized to *Gapdh* and *R18s* (n=4–6). **(H)** Safranin-O (red)/fast green (blue)/hematoxylin(purple)-stained tissue sections of miPSC derived cartilage co-cultured with NF-κB-LUC or NF-κB-IL-1Ra macrophages. **(I)** Type II collagen tissue sections (brown stain) of miPSC derived cartilage co-cultured with NF-κB-LUC or NF-κB-IL-1Ra macrophages. Statistical significance were assessed using a two-way ANOVA with Tukey’s post hoc test; values represent mean with standard error of the mean (SEM) (n = 4–6).

To determine if the protective capacity of NF-κB-IL-1Ra-macrophages was also preserved during indirect interactions, we utilized transwell co-cultures with cartilage pellets. Pellets were pretreated with 0, 0.5, or 1 ng/mL IL-1β for 24 hours before being placed in transwell co-culture with either NF-κB-LUC or NF-κB-IL-1Ra macrophages and restimulated with 0, 0.5 or 1 ng/mL IL-1β, respectively (Figure 3A). All cultures contained significant amounts of IL-1Ra following IL-1β treatment (Figure 3B, Supplemental Figure 3A). Co-cultures with NF-κB-IL-1Ra macrophages produced significantly higher levels of IL-1Ra than NF-κB- LUC macrophages, secreting 50 pg/10^3^ macrophages and 100 pg/10^3^ macrophages of IL-1Ra by 48 hours in response to 0.5 ng/mL and 1 ng/mL of IL-1β, respectively. NO levels were reduced in NF-κB-IL-1Ra macrophage co-cultures following 1 ng/mL IL-1β treatment; however, there were no significant differences between co-cultures at 0 and 0.5 ng/mL IL-1β (Figure 3C). Changes in gene expression were examined for expression factors pertinent to both pellets (Figure 3F) and engineered macrophages (Figure 3G). *Acan* and *Col2a1* were reduced and inflammatory genes *Ccl2, Il6*, *Mmp13*, and *Pges2* were upregulated to similar extents for all groups following IL-1β treatment. While inflammatory gene expression was reduced for pellets in NF-κB-IL-1Ra macrophage co-cultures, there were no statistically significant differences in macrophage inflammatory gene expression between NF-κB-LUC and NF-κB-IL-1Ra macrophage co-cultures following 48 hours (Figure 3G); however statistically significant differences were present in monoculture between NF-κB-LUC and NF-κB-IL-1Ra macrophages (Supplemental Figure 3B).

Co-culture with NF-κB-IL-1Ra macrophages exhibited a marginal increase in sGAG/DNA content in pellets administered 0 and 0.5 ng/mL of IL-1β over those co-cultured with NF-κB-LUC macrophages (Figure 3D). There was no significant difference in sGAG/DNA content in pellets treated with 1 ng/mL of IL-1β or secretion into the media (Figure 3D, E and H). All non-treated pellets showed rich Safranin-O staining for sGAG (Figure 3H). Pellets treated with IL-1β displayed less Safranin-O staining that was further reduced during co-culture with NF-κB-LUC macrophages in a dose dependent manner and was protected in co-cultures with NF-κB-IL-1Ra macrophages. Type II collagen demonstrated a similar dose-dependent reduction in histological staining in pre-treated pellets (Figure 3I). When co-cultured with NF-κB-LUC macrophages, this collagen expression was further reduced during co-culture with IL-1β. Conversely, NF-κB-IL-1Ra macrophage co-culture demonstrated a chondroprotective effect on the pellets with minimal loss of staining observed following IL-1β treatment.

### NF-κB-IL-1Ra macrophages offer similar protection against IL-1-driven matrix degradation as anakinra

**Figure 4:**
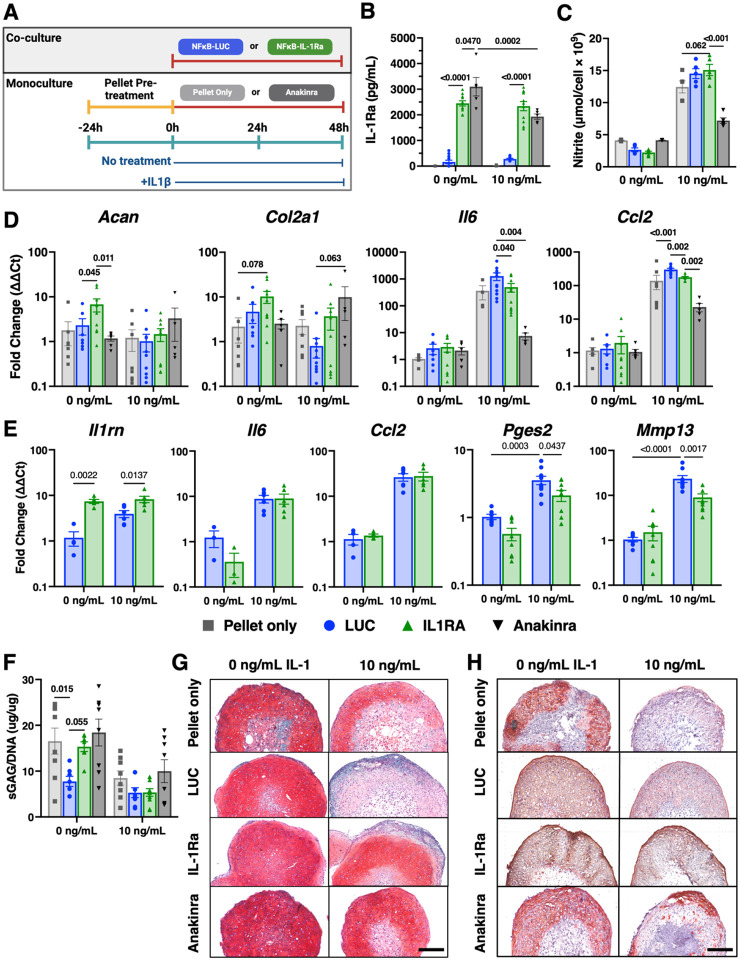
NF-κB-IL-1Ra macrophages offer moderate chondroprotection against inflammatory mediators and inflammatory driven matrix degradation in response to IL-1β. **(A)** Timeline of co-culture experimental conditions. miPSC derived cartilage pellets were initially pre-treated with 0 or 10 ng/mL of IL-1β for 24 hours. Pellets were then placed into new monoculture wells with 0 or 1 mg/mL of anakinra or in co-culture with transduced macrophages (NF-κB-LUC or NF-κB-IL-1Ra) and again treated with 0 or 10 ng/mL of IL-1β for an additional 48 hours in a 24 well transwell plate (n=6). **(B)** IL-1Ra levels in the media following 48 hours of monoculture or co-culture, as measured by ELISA. **(C)** Concentration of nitric oxide secreted into the media normalized to cell number following treatment with 0 or 10 ng/mL of IL-1β. **(D)** Gene expression of miPSC derived cartilage pellets in mono- and co-culture in reference to non-treated control pellets and **(E)** engineered macrophages in reference to a non-treated control when challenged with IL-1β for 48h normalized to *Gapdh* and *R18s* (n=4–6).**(F)** Amount of sGAG per pellet normalized to DNA content. **(G)**Safranin-O (red)/fast green(blue)/hematoxylin(purple)-stained tissue sections of miPSC derived cartilage; scale bar = 200mm. **(H)** Type II collagen tissue sections (brown stain) of miPSC derived cartilage; scale bar = 200mm. Statistical significance was assessed using a two-way ANOVA with Tukey’s post hoc test; values represent mean with standard error of the mean (SEM) (n = 4–6).

We next wanted to evaluate the potential of our therapeutic NF-κB-IL-1Ra macrophages to a comparable treatment used in the clinic, anakinra (anakinra, Sobi AB, Stockholm, Sweden), which has been used for the treatment of inflammatory arthritis. Pellets were pre-treated with either 0 or 10 ng/mL IL-1β for 24 hours. Pellets were either maintained in monoculture and administered additional 0 or 10 mg/mL of anakinra, the highest clinically recommended dose (mg/kg) or placed in transwell co-culture with NF-κB-LUC or NF-κB-IL-1Ra macrophages before restimulation with 0 or 10 ng/mL IL-1β (Figure 4A) [51].

Following co-culture, NF-κB-IL-1Ra macrophages produced significantly more IL-1Ra than NF-κB-LUC macrophages; however, there was no difference in IL-1Ra produced when macrophages were co-cultured with 0 or 10 ng/mL IL-1β (Figure 4B). Anakinra pellet cultures had significantly higher IL-1Ra levels remaining than NF-κB-IL-1Ra macrophage co-cultures with no treatment. However, when treated with IL-1β, pellets cultured in the presence of anakinra or co-cultured with NF-κB-IL-1Ra macrophages possessed similar amounts of remaining IL-1Ra. NO levels in non-treated cultures were relatively low for all groups. However, following IL-1β treatment, NO was significantly reduced in anakinra pellet cultures while there was no notable reduction in NO in macrophage co-cultures (Figure 4C).

While untreated pellets co-cultured with NF-κB-IL-1Ra macrophages significantly upregulated gene expression for *Acan* and *Col2a*, IL-1β treatment abrogated the upregulation (Figure 4D). Co-cultures with NF-κB-LUC macrophages led to the largest downregulation in matrix gene expression when treated with IL-1β (Figure 4D). While anakinra-treated pellet cultures were significantly protected against upregulation of inflammatory mediators *Il6* and *Ccl2* following IL-1β treatment, NF-κB-IL-1Ra macrophage co-cultures also demonstrated protection, albeit at reduced fold changes in upregulation as compared to pellet monocultures and NF-κB-LUC macrophage co-cultures (Figure 4E). Pellets co-cultured with NF-κB-LUC macrophages exhibited the highest level of upregulation in inflammatory gene expression of any condition. While there was no downregulation in *Ccl2* or *Il6* in NF-κB-IL-1Ra macrophages compared to NF-κB-LUC macrophages in co-cultures, there was significant downregulation in the gene expression of the *Pges2* and *Mmp13*, which may be mediated through the increase in *Il1rn* expression (Figure 4E).

There was no significant difference in sGAG/DNA content between pellets in any condition following IL-1β treatment though anakinra pellets demonstrated the highest sGAG/DNA ratio (Figure 4F). In non-treated conditions, both pellets administered anakinra or co-cultured with NF-κB-IL-1Ra macrophages exhibited similar levels of sGAG/DNA as that of the pellet monocultures while pellets co-cultured with NF-κB-LUC macrophages demonstrated a significant reduction in sGAG/DNA. All pellets exhibited high levels of Safranin-O staining in non-treated conditions. With treatment of 10 ng/mL of IL-1β, Safranin-O staining was significantly reduced in the NF-κB-LUC macrophage co-culture and to a lesser extent in pellet monoculture controls. Pellets co-cultured with anakinra and NF-κB-IL-1Ra macrophages were protected from loss of Safranin-O staining (Figure 4G). With no treatment, type II collagen staining did not change significantly in any condition (Figure 4H). Following IL-1β treatment, expression of type II collagen was significantly reduced in the pellet monocultures and NF-κB-LUC macrophage co-cultures; however, type II collagen expression was moderately protected in pellets cultured with anakinra and fully protected in pellets co-cultured with NF-κB-IL-1Ra macrophages.

### NF-κB-IL-1Ra macrophages inhibit inflamed synovial fibroblasts from inflammatory activation in response to IL-1β

**Figure 5:**
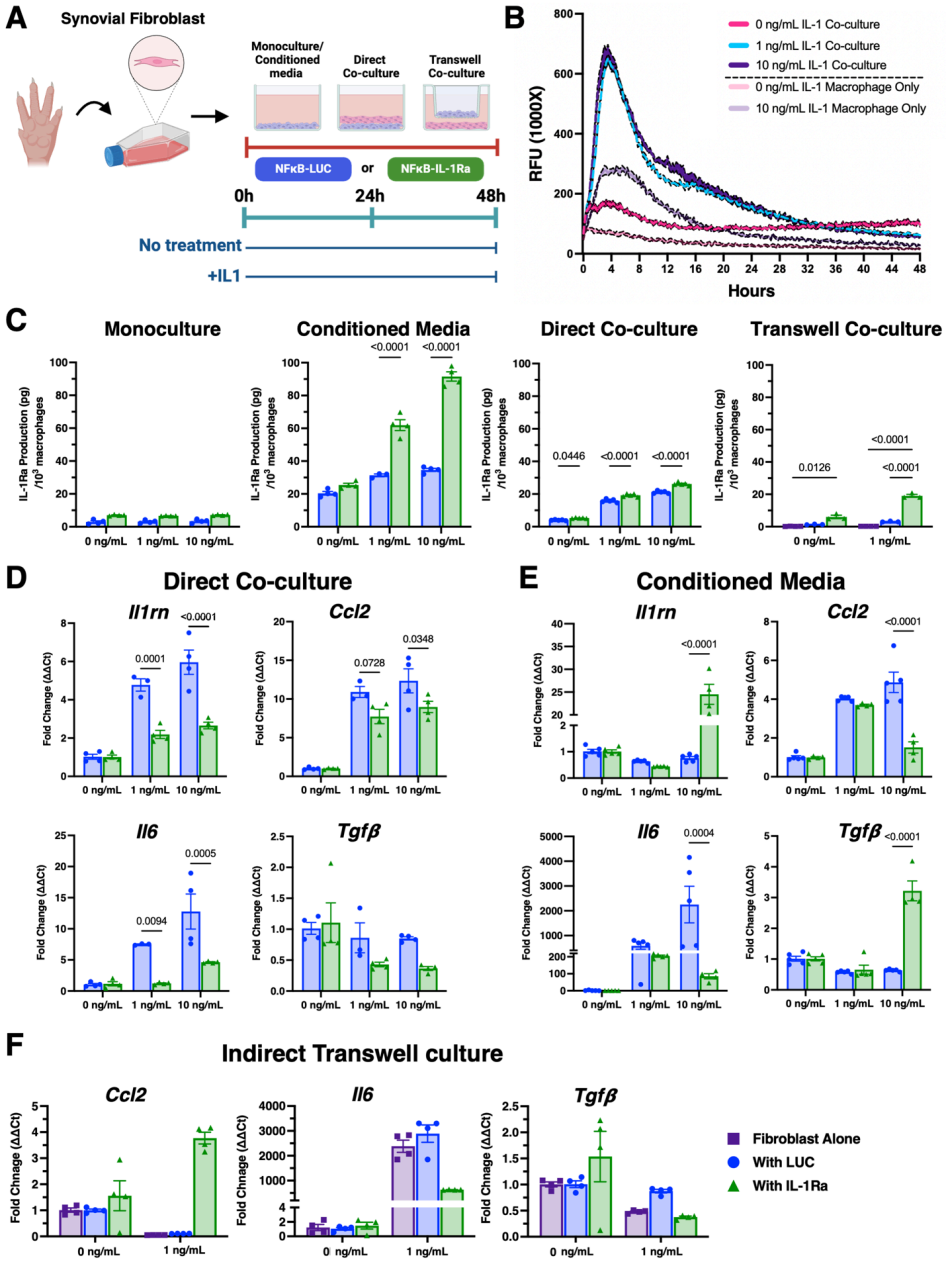
NF-κB-LUC and NF-κB-IL-1Ra macrophages differentially activate to co-culture with synovial fibroblasts following an inflammatory stimulus. **(A)** Timeline of co-culture experimental conditions. **(B)** NF-κB-LUC macrophages demonstrate circuit activation by 4 hours following treatment with IL-1β that significantly increases in response to co-culture with synovial fibroblasts (n=6). **(C)** IL-1Ra production increased in response to IL-1b treatment with indirect and conditioned media demonstrating higher levels of production between control and therapeutic macrophages (n=3–5). **(D-F)** Gene expression of NF-κB-IL-1Ra and NF-κB-LUC macrophages with synovial fibroblasts when challenged with IL-1β for 48 hours normalized to *Gapdh* and *R18s* (n=4) demonstrated significant reduction in NF-κB responsive genes when co-cultured with NF-κB-IL-1Ra macrophages. Data was assessed using a two-way ANOVA with Tukey’s post hoc test; values represent mean with standard error of the mean (SEM) (n = 4–6).

We then utilized the K/BxN serum transfer arthritis (STA) mouse model to induce rapid and robust arthritis in naïve mice to better understand the interplay between the NF-κB-IL-1Ra macrophages and synovial fibroblasts, a prominent cell population that influences macrophage phenotype in RA [48, 49]. We examined the direct and indirect response of engineered macrophages on synovial fibroblasts isolated from the paws of K/BxN STA mice at day 7, the peak of the disease in this model (Figure 5A). To examine the role of synovial fibroblasts on circuit activation, NF-κB-LUC macrophages in monoculture were compared to NF-κB-LUC macrophages placed in direct co-culture with isolated synovial fibroblasts. Cells were incubated with luciferin, recorded continuously for 24 hours to establish a baseline luminescent signal, and then treated with IL-1b (10 ng/mL) and monitored for 48 hours (Figure 5B). NF-κB-LUC macrophages co-cultured with synovial fibroblasts had higher circuit activity (~2.5 times more) and peaked earlier (~4–8 hours) than monocultured cells, but all returning to baseline by 44 hours after stimulation.

To examine the impact of fibroblast-macrophage crosstalk on circuit activation and inflammatory mitigation, NF-κB-LUC and NF-κB-IL-1Ra macrophages were either treated with fibroblast conditioned media or placed into direct or indirect (transwell) co-culture with synovial fibroblasts prior to treatment with 0, 1, or 10 ng/mL of IL-1b. NF-κB-IL-1Ra macrophages produced significant amounts of IL-1Ra in response to both direct culture with synovial fibroblasts as well as conditioned media from synovial fibroblasts that was comparable to co-culture with miPSC-derived cartilage (Figure 5C). When inflammatory activation was examined at the gene level, macrophages in direct co-culture with synovial fibroblasts exhibited a much higher inflammatory response than those treated with conditioned media (Figure 5D and E). Similar to chondrocyte pellet co-cultures, there was no protection observed at low levels of IL-1b. However, significant downregulation of inflammatory genes *Ccl2* and *Il6* and upregulation of *Il1rn* and *Tgfb* was observed in response to co-culture with NF-κB-IL-1Ra macrophages (Figure 5D). Though macrophages cultured in conditioned media had less overall activation, NF-κB-IL-1Ra macrophages were observed to downregulate inflammatory genes similarly to other culture conditions, at both levels of IL-1b treatment (Figure 5E).

In transwell co-cultures of synovial fibroblasts and engineered macrophages, there was no significant difference in IL-1Ra production by NF-κB-IL-1Ra macrophages between direct and transwell co-culture (Figure 5C). However, NF-κB-LUC macrophages produced significantly less IL-1Ra in transwell co-cultures suggesting that direct contact might be necessary to initiate optimal macrophage production of IL-1Ra. The amount of IL-1Ra produced by NF-κB-IL-1Ra macrophages was sufficient to moderate the inflammatory activation, as evidenced by *Il6* downregulation in NF-κB-LUC macrophage co-cultures (Figure 5F).

### NF-κB-IL-1Ra macrophages persist following injection in the K/BxN serum transfer mouse model of RA

**Figure 6:**
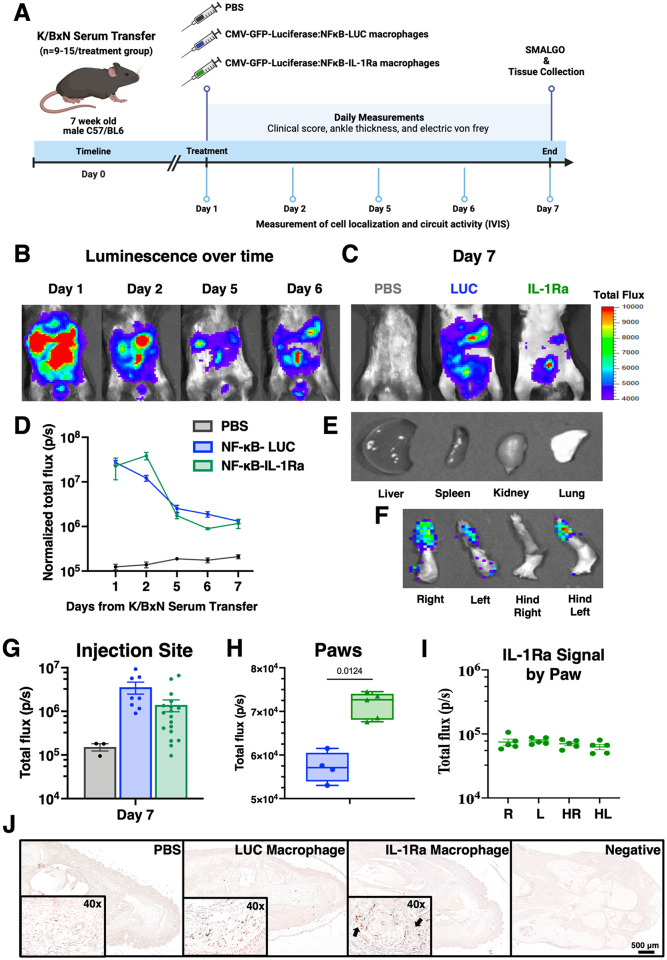
NF-κB-IL-1Ra engineered macrophages maintain presence for at least 7 days in the K/BxN serum transfer mouse model of RA. **(A)** Mice injected with NF-κB-IL-1Ra macrophages exhibited reduced **(B/F)** Bioluminescent imaging over time following injection of NF-κB-IL-1Ra and NF-κB-LUC macrophages in K/BxN mice demonstrated a reduction over time but as of day 7 there was still significant macrophage presence at the injection site **(C/G)** with **(D, H, I)** presence in the paws but **(E)** no presence in the clearance organs but. **(J)** Immunohistochemistry for GFP^+^ staining confirmed injected macrophage presence in the paws of mice compared to non-treated and negative stained sections. Values represent mean with SEM (n=9–15); photons/second (p/s) with a baseline of 10^4^; IP intraperitoneal; R, right; L, left; HR, hind right; HL, hind left.

The K/BxN STA mouse model was again utilized to induce rapid and robust arthritis in naïve mice to examine disease mediators relevant in RA [48, 49]. We examined the therapeutic potential of our NF-κB-IL-1Ra engineered macrophages to mitigate disease activity in this model [52]. Twenty-four hours following STA initiation, mice were intraperitoneally (IP) administered a PBS control, a vehicle control (NF-κB-LUC macrophages), or therapeutic (NF-κB-IL-1Ra macrophages) (Figure 6A). To track macrophages *in vivo*, cells were transduced with a constitutive CMV-GFP-Luciferase circuit in addition to the corresponding self-regulating NF-κB dependent circuit. Luminescence was measured one hour following injection to confirm successful delivery and was quantified daily to observe macrophage migration and clearance. High total luminescent signal was detected in mice one hour following IP delivery and was present, although diminishing in intensity, until day 4 following injection at which point macrophage presence rapidly decreased but persisted above baseline (Figure 6B and F). By day 7, luminescence levels in both NF-κB-LUC and NF-κB-IL-1Ra injected mice were significantly diminished (Figure 6C and G). Mice receiving NF-κB-LUC macrophages exhibited a higher luminescent signal than NF-κB-IL-1Ra macrophages that was driven by the presence of both the constitutive and NF-κB luciferase circuits, where the NF-κB-inducible circuit likely produced an additive signal in response to increased levels of inflammation in the K/BxN mice. There was little signal for all mice at day 7 in typical clearance organs such as the kidneys, spleen, liver, or lungs (Figure 6D). When examining for macrophage presence in joints *ex vivo*, 5 out of 12 NF-κB-IL-1Ra macrophage-treated mice expressed a low but definitive luminescence signal in their paws; however, no NF-κB-LUC macrophage-treated mice possessed luminescence signal in their paws (Figure 6E and H). Immunohistochemistry for GFP^+^ staining confirmed presence of IL-1Ra macrophage in the paws of treated mice (Figure 6I).

### Engineered macrophages polarize to an inflammatory phenotype following injection into K/BxN mice

**Figure 7:**
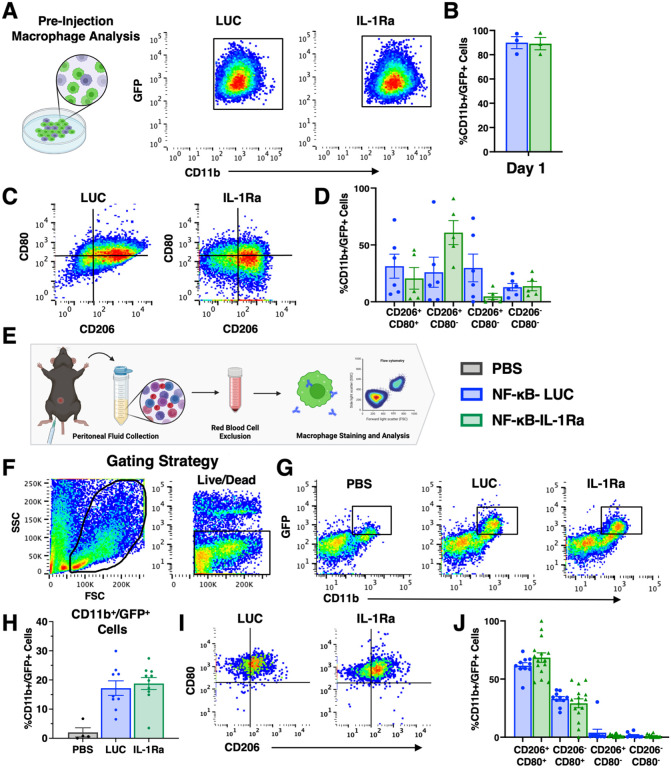
Engineered macrophages polarize following injection to a more inflammatory phenotype in the K/BxN treated mice. **(A and B)** Prior to injection macrophage efficiency was profiles using flow cytometry for positive CD11b and GFP labeling. **(C and D)**Macrophage activation was also examined for inflammatory marker CD80 and anti-inflammatory marker 206. At 6 days following injection, peritoneal fluid at injection site was collected and examined to observe **(E-H)** presence of GFP positive macrophages still present at the injection site as well as **(I and J)** macrophage activation post injection using the same markers CD80 and CD206.

Engineered macrophages were profiled prior to injection in terms of transfection efficiency and phenotype. Following transfection and differentiation, both NF-κB-IL-1Ra and NF-κB-LUC macrophages were fully differentiated (94.6 and 90.9% CD11b^+^, respectively) and demonstrated a transfection efficiency of around 90–95% (Figure 7A and B). Both macrophage populations demonstrated high CD206^+^ and moderate CD80^+^ expression, with NF-κB-LUC macrophages showing a slightly higher percentage of double positive CD206/CD80 population than NF-κB-IL-1Ra macrophages (Figure 7C and D). At sacrifice, macrophages still present the peritoneal cavity were isolated and profiled (Figure 7E).

Mice that had no IVIS signal were excluded from analysis (Figure 7F and G). Injected macrophages were identified by CD11b/GFP^+^ expression and dead cells were removed prior to profiling (Figure 7F–H). Following injection, both NF-κB-LUC and NF-κB-IL-1Ra macrophages from treated mice significantly polarized to a more inflammatory state, as evidenced by the loss of the CD206^+^/CD80^−^ population that was present before injection (Figure 7I and J).

**Figure 8:**
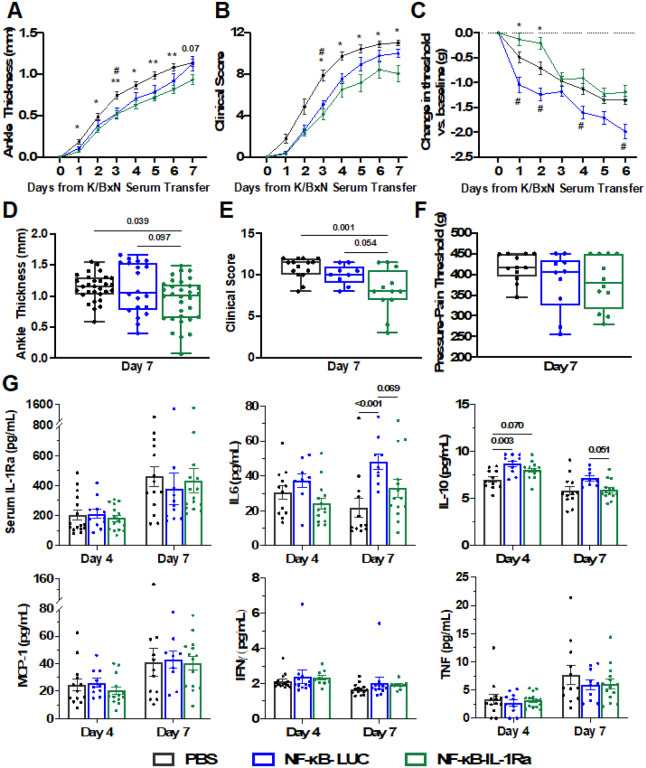
NF-κB-IL-1Ra engineered macrophages mitigate disease in the K/BxN serum transfer mouse model of RA. Mice injected with NF-κB-IL-1Ra macrophages exhibited reduced (A) ankle thickness (B) clinical scores and (C) pain overtime in comparison to PBS control animals. By day 7, while this significance was pronounced between all groups in terms of (D) ankle thickness and (E) clinical scores, (F) pain measurement by SMALGO was not significantly different from baseline. (G) IL-1Ra present in the serum of mice was not significantly different by day 7 but mice that received NF-κB-IL-1Ra macrophages still showed less systemic inflammation as compared to mice with NF-κB-LUC macrophages or PBS controls. Data was assessed using a one or two-way ANOVA with Tukey’s post hoc test for * NF-κB-IL-1Ra vs PBS and # NF-κB-LUC vs PBS; * p < 0.05, ** p < 0.01, *** p < 0.001, **** p < 0.0001; values represent mean with SEM (n=9–15).

Following treatment with NF-κB-IL-1Ra macrophages, mice demonstrated significant amelioration in disease severity, as evidenced by reduction in ankle thickness and clinical scores compared to both NF-κB-LUC treated and PBS control animals (Figure 8A and B). Significant mitigation was maintained at day 7 with NF-κB-IL-1Ra macrophage-treated animals exhibiting 25.8% less clinical severity (Figure 8D and E). Compared to PBS treated control animals, NF-κB-IL-1Ra macrophage-treated mice had decreased mechanical pain sensitivity, as measured by electronic Von Frey pain tests, while NF-κB-LUC macrophage-treated mice had increased pain sensitivity (Figure 8C and F). While IL-1Ra measured in the serum was the same for all treatment groups (Figure 8G), mice that received NF-κB-IL-1Ra macrophages showed decreased IL-6 and increased IL-10 cytokine levels in the serum as compared to NF-κB-LUC macrophage-treated or PBS control animals.

**Figure 9:**
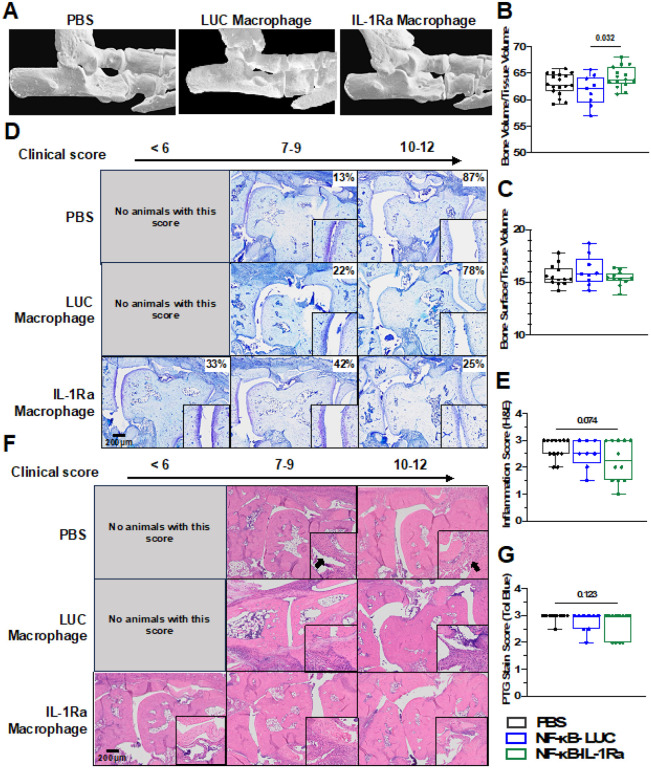
IL-1Ra engineered macrophages protect against bone damage. **(A)** Region of micro-CT analysis for all mice examining **(B)** Bone volume/tissue volume and **(C)** bone erosion as measured by bone surface/bone volume. At day 7, NF-κB-IL-1Ra macrophages somewhat reduced cartilage degradation **(D/E)** and inflammation **(F/G)** correlating to clinical score. Total animals per group in each range of clinical score is displayed as a percentage. Zoomed images are shown in the black boxes. Data was assessed using a one-way ANOVA with Tukey’s post hoc test; values represent mean with SEM (n=9–15).

Micro-computed tomography (microCT) of hind paws in mice receiving NF-κB-IL-1Ra injections displayed higher bone volume/tissue volume (BV/TV) compared to mice receiving NF-κB-LUC macrophages. However, no differences in bone surface density (BS/TV), a measurement of bone erosion, were observed between any groups (Figure 9A–C). Furthermore, additional histological analysis showed cartilage, bone, and proteoglycan degradation as well as hallmarks of inflammation present in all animals (Figure 9D and F). While there was no statistically significant difference in cartilage loss or inflammation across all treatment groups, protection from cartilage and bone loss and decreased inflammation scores were observed in some NF-κB-IL-1Ra macrophage-treated mice (Figure 9E and G).

## Conclusions

A significant challenge for the treatment of RA has been the delivery of appropriate levels of anti-cytokine biologic drugs to inhibit pathologic inflammation while avoiding excessive levels of drug that lead to suppression of the immune system and adverse events. In this regard, we developed a macrophage-based cell therapy to allow for cell homing and engraftment to sites of inflammation and self-regulating drug production that is proportional to the level of inflammation, circumventing the need for frequent, systemic dosing with anti-inflammatory biologics. Macrophages have fundamental characteristics of homing and engraftment to sites of inflammation and play critical roles in orchestrating immune responses; therefore, they represent a viable cell vehicle for targeting a variety of diseases, particularly autoimmune conditions. Indeed, macrophages have been successfully used as a system for the delivery of both anti-tumorigenic and bacterial agents to mitigate disease [53]. Here, we demonstrated a proof of concept for engineered macrophage systems capable of sensing and dynamically responding to inflammation by producing an anti-inflammatory mediator in a self-regulating manner. These engineered macrophages were able to repeatably produce therapeutic levels of IL-1Ra in response to multiple inflammatory stimuli to moderate their own response as well as protect their local environment. Additionally, these macrophages were able to remain engrafted *in vivo* for up to one week suggesting the potential for more prolonged therapeutic effects as compared to standard stem cell therapies, which are generally cleared within 24–48 hours [54].

Using lentiviral transduction, we engineered primary BMDMs with an NF-κB-responsive element that produced IL-1Ra (or luciferase control) downstream of an IL-1 stimulus. As the clinical course of RA is typically punctuated by flares alternating with remissions, an ideal therapeutic strategy should be responsive and effective in dampening inflammation. Both systems responded rapidly to physiologic and supraphysiologic doses of IL-1, peaking by 12 hours following treatment. Importantly, while engineered macrophages continuously produced a basal level of IL-1Ra, only with cytokine stimulation were clinically relevant levels of IL-1Ra produced. Both circuits activated in a dose-dependent manner that self-attenuated by 48 hours and was repeatable across multiple stimulations. Therefore, this system offers an attractive source of IL-1Ra production that can be repeatedly triggered to a comparable magnitude and turned “off” when not stimulated, representing advantages that could mitigate adverse events associated with continuous biologic administration.

To assess the dynamic activity of the gene circuit, multiple conditions were analyzed to reflect potential microenvironments of an RA joint from a moderately inflamed joint to the highly inflammatory conditions during a flare with actively inflamed cartilage and synovial tissue and fluid. Both conditions were compared to groups that did not receive an external inflammatory stimulus in co-culture. IL-1β has been found to be elevated in the synovial fluid and plasma of RA patients and is associated with increased production of pro-inflammatory mediators including cytokines, chemokines, prostaglandins, and NO, as well as matrix metalloproteinases (MMPs) and a disintegrin and metalloproteinase with thrombospondin motifs (ADAMTS) in articular cartilage [55, 56]. Therefore, IL-1β was used as the primary cytokine stimulus in mono- and co-cultures. However, supplementing culture media with IL-1β, IL-1α, or TNF-α all induced a relevant phenotype in the cartilage pellets similar to an RA-like joint with increased expression of inflammatory genes *Il6* and *Ccl2*, reduced expression of matrix genes *Acan* and *Col2a1*, and reduced histological Safranin-O staining, supporting their use as an *in vitro* model suitable for characterizing the effects of therapeutic macrophages.

Engineered macrophages co-cultured with cartilage pellets responded similarly to macrophage monoculture where macrophages produced significant amounts of IL-1Ra in response to IL-1β that was able to offer some level of chondro-protection. However, there was a clear distinction between direct and indirect co-culture groups as well as low and high levels of IL-1β in co-culture in terms of therapeutic mitigation. Studies have demonstrated that production of IL-1Ra, a natural inhibitor of IL-1, is elevated in inflammatory RA joints [57]; however, the levels produced in RA joints are usually insufficient to reduce damage, as a concentration of 10-to-100-fold excess of IL-1Ra is required to effectively inhibit IL-1, which is a limiting factor for IL-1Ra as a clinical therapeutic [58]. Stimulation with 1 or 10 ng/mL IL-1β used in these experiments represent super-physiological levels as concentrations of IL-1β have been reported between 0.115 to 0.5 ng/mL in the synovial fluid in early spontaneous joint degeneration [55]. miPSC-cartilage pellets were significantly more sensitive at these lower levels of IL-1 than macrophages which have been shown to require higher doses to activate to a similar level [42, 59]. From the monoculture characterization of the circuit activation in macrophages, these differences are not surprising as peak activation was shown to be observed between 5–10 ng/mL of IL-1 which suggests that the engineered macrophages were not fully activating to protect pellets during the low dose transwell experiment. Notably, NF-κB-IL-1Ra macrophages performed similarly to anakinra *in vitro*, suggesting that on-demand cell-based release of IL-1Ra at therapeutic levels offers equivalent therapeutic protection with a superior mode of delivery, as compared to the standard exogenous delivery of anakinra (e.g., twice daily subcutaneous injections).

In RA, macrophages respond to inflammatory activation by synovial fibroblasts by both direct and indirect interactions in the synovial lining and fluid [6, 60]. When we examined the effects these dynamics had on the activation of the NF-κB circuits, both NF-κB-LUC and NF-κB-IL-1Ra macrophages had significantly higher responses to direct co-cultures than either indirect or conditioned media alone. This is unsurprising, as recent studies have focused on the importance of synovial broblast-macrophage mechanical cues for the propagation of inflammatory signaling. Despite the lower activation in transwell and conditioned media cultures, NF-κB-IL-1Ra macrophages were still able to protect both their own and the inflammatory activation of synovial fibroblasts in co-culture for all types of macrophage-fibroblast crosstalk.

A cell-based drug delivery system that has the potential to remain for a significant time following injection in the body could overcome the burden of repeated self-administered injections. Our studies show that once injected, macrophages remain both activated and present for up to 7 days in multiple mouse models, suggesting they have potential as a cell-based drug delivery system to alleviate injection burden (Fig. 6B and Supplemental Fig. 4A-D). The ability of NF-κB-IL-1Ra macrophages to dynamically sense and regulate the highly inflammatory environment of the K/BxN STA model to mitigate both inflammation and structural damage demonstrated the therapeutic potential of this approach. While NF-κB-IL-1Ra macrophages were not successful in treating all mice, it demonstrated a similar effect to the clinical response of anakinra, which is unable to fully mitigate disease, where only ~ 40% of patients have a moderate response [61]. While some change in phenotype was expected, the degree to which macrophage phenotype following injection polarized to a more inflammatory state in the K/BxN STA mice was unexpected as similar injections into both control and an acute joint injury model did not elicit as significant a phenotype shift (Supplementary Fig. 4H and I). These results demonstrate the need for further investigation to tailor potential modifications to disease specific activity when to mediate macrophage-host response.

Immunoengineered macrophages have been a leading target in recent years due to their highly influential role in many diseases from musculoskeletal diseases like RA and osteoarthritis (OA) to different forms of cancer. Early methods to mediate the chronic inflammatory environment of RA effectively used genetic modification of macrophage phenotype to guide downstream cytokine signaling during inflammation. Other techniques such as the attachment or encapsulation of polymer materials and drug reservoirs to macrophages have been very successful [40, 41]. For example, macrophages with engineered biomaterial cytokine reservoirs can be employed in the context of OA and RA as a precise tool for cell-specific biomaterial-based mediation of inflammation. However, macrophages act as both anti- and pro-inflammatory mediators, and as such, it is important to bear in mind the off-target effects genetic modifications might have on functions outside of their disease target. Thus, the development of a more precise and disease-specific cell therapy that meets this challenge is crucial for designing more effective interventions.

Our engineered macrophage therapy offers a novel solution to this challenge by homing to the site of inflammation, persisting significantly longer biologically than conventional IL-1Ra dosage methods used clinically, and sensing and responding to inflammation with a therapeutic level of biologic drug in a self-regulating manner. Indeed, our engineered macrophages successfully produced significant amounts of IL-1Ra that initiated anti-inflammatory and chondroprotective effects, while not appreciably modulating overall macrophage phenotype or function and maintain their presence upon injection. While we utilized IL-1Ra to characterize their potential, this system could be made applicable for a wide range of cytokine treatments that would benefit from a macrophage-based cell therapy with the ability to both home to regions of inflammation and self-regulate protein delivery. The continued develop of these cell-based immunoengineering strategies for localized and self-regulated drug delivery may provide novel therapeutics to more targeted and effective treatments in a wide range of immunological diseases.

## Data Availability

All data underlying the figures are available upon request.
